# ID 118 - Desenvolvimento e Aplicação de Curativo à Base de Látex (*Hevea brasiliensis*) Contendo Curcumina e Papaína Lipossomal Associadas à LEDterapia para Tratamento de Feridas Diabéticas

**DOI:** 10.5327/2237-9622.2025.v34s1.118

**Published:** 2025-11-25

**Authors:** Franciéle de Matos da Silva, Jaqueline R. da Silva, Wellington Rodrigues, Breno Amadeus S. M. Sousa, Suélia Siqueira R. F. Rosa, Marcella Lemos B. Carneiro

**Keywords:** feridas diabéticas, curcumina, papaína, LEDterapia, biomembranas, látex

## Abstract

**Introdução:**

As feridas diabéticas, uma complicação comum do diabetes mellitus, representam um dos principais desafios no tratamento de pacientes com essa doença crônica. Caracterizadas pela dificuldade de cicatrização, essas feridas estão frequentemente associadas a fatores como neuropatia, doença vascular periférica e uma resposta inflamatória comprometida. As complicações incluem infecções recorrentes, úlceras crônicas, gangrena e, nos casos mais graves, a amputação de membros. Essas consequências geram um impacto significativo na qualidade de vida dos pacientes e no sistema de saúde, com custos elevados para o tratamento e hospitalizações prolongadas. O custo do tratamento de feridas diabéticas no Brasil é elevado, sobrecarregando o Sistema Único de Saúde (SUS) com hospitalizações prolongadas, cirurgias e cuidados intensivos. A necessidade de tecnologias inovadoras e de baixo custo para o tratamento de feridas diabéticas é, portanto, urgente. Nesse contexto, foi desenvolvido um curativo à base de látex () contendo curcumina e papaína lipossomal, combinado com um dispositivo de LED de comprimento de onda variado (450±20 nm à 636±20 nm) emissor de luz vermelha de baixa intensidade denominado RAPHA©, uma tecnologia inovadora a de otimização do processo de neoformação tecidual. Essas novas abordagens buscam não apenas reduzir os custos associados ao tratamento, mas também proporcionar alternativas acessíveis e eficazes para populações. O desenvolvimento de terapias acessíveis e de alta eficácia é crucial para minimizar as complicações e os custos associados às feridas diabéticas, além de melhorar a qualidade de vida dos pacientes.

**Método:**

Biomembranas foram confeccionadas a partir de látex diluído em água, combinado com soluções lipossomais, moldadas em formas de poliestireno e cortadas em quadrados de 2 x 2 cm. Os testes in vivo utilizaram ratos Wistar machos com diabetes induzido por aloxana monoidratada e feridas de 6 mm no dorso. Foram formados cinco grupos experimentais: (1) controle sem tratamento, (2) látex com curcumina lipossomal + LED, (3) látex com papaína lipossomal + LED, (4) látex isolado + LED, e (5) látex com curcumina e papaína lipossomal + LED. Os tratamentos foram aplicados a cada 48 horas durante 11 dias, com exposição à LED (RAPHA©) por 10 minutos. O tamanho das feridas e o perfil diabético dos animais foram avaliados, e os dados foram submetidos à ANOVA one-way e pós-teste de Tukey (p < 0,05).

**Resultados:**

Os animais diabéticos apresentaram hiperglicemia, poliúria, polidipsia e perda de peso. Biomembranas com curcumina e papaína não alteraram as propriedades estruturais do látex. Os grupos tratados com LEDterapia associada a biomembranas contendo curcumina e papaína mostraram fechamento de 99% e 95% das feridas, respectivamente.

**Conclusão:**

A associação de curcumina e papaína ao látex, aliada à LEDterapia, é promissora no tratamento de feridas diabéticas, acelerando a cicatrização.

